# *XRCC1* gene polymorphisms in a population sample and in women with a family history of breast cancer from Rio de Janeiro (Brazil)

**DOI:** 10.1590/S1415-47572009000200008

**Published:** 2009-06-01

**Authors:** Priscila Falagan-Lotsch, Marina S. Rodrigues, Viviane Esteves, Roberto Vieira, Luis C. Amendola, Dante Pagnoncelli, Júlio C. Paixão, Claudia V. De Moura Gallo

**Affiliations:** 1 Departamento de Biologia Celular e Genética, Instituto de Biologia Roberto Alcântara Gomes, Universidade Estadual do Rio de Janeiro, Rio de Janeiro, RJBrazil; 2 Departamento de Ginecologia, Instituto Fernandes Figueira, Fundação Oswaldo Cruz, Rio de Janeiro, RJBrazil; 3 Departamento de Biologia Molecular, Instituto Fernandes Figueira, Fundação Oswaldo Cruz, Rio de Janeiro, RJBrazil

**Keywords:** *XRCC1 gene polymorphisms*, breast cancer susceptibility, Brazilian population

## Abstract

The X-ray repair cross-complementing Group1 (*XRCC1)* gene has been defined as essential in the base excision repair (BER) and single-strand break repair processes. This gene is highly polymorphic, and the most extensively studied genetic changes are in exon 6 (Arg194Trp) and in exon 10 (Arg399Gln). These changes, in conserved protein sites, may alter the base excision repair capacity, increasing the susceptibility to adverse health conditions, including cancer. In the present study, we estimated the frequencies of the *XRCC1* gene polymorphisms Arg194Trp and Arg399Gln in healthy individuals and also in women at risk of breast cancer due to family history from Rio de Janeiro. The common genotypes in both positions (194 and 399) were the most frequent in this Brazilian sample. Although the 194Trp variant was overrepresented in women reporting familial cases of breast cancer, no statistically significant differences concerning genotype distribution or intragenic interactions were found between this group and the controls. Thus, in the population analyzed by us, variants Arg194Trp and Arg399Gln did not appear to have any impact on breast cancer susceptibility.

Rio de Janeiro is the Brazilian State with the highest number of breast cancer cases: 92.77 cases/10^5^ women, according to INCA (2008) estimates. Many risk factors are involved in breast cancer, the genetic component being one of the most important affecting its frequency (Antoniou and Easton, 2006). Therefore, at the population level, breast cancer shows a high degree of familial aggregation, in which 50% of the cases are explained by genes mainly involved in maintaining genome integrity (Walsh and King, 2007). DNA damage plays a central role in carcinogenesis and, consequently, genes involved in DNA repair are considered key genes in cancer development. The X-ray repair cross-complementing Group1 (*XRCC1*) gene has been defined as essential in the base excision repair (BER) and single-strand break repair processes (Brem and Hall, 2005). The importance of this gene is highlighted by the description of its null mutant mice, whose embryonic development is arrested (Tebbs *et al.*, 1999). The *XRCC1* gene codes for a protein that is capable of interacting with different repair proteins. More than 60 validated single-nucleotide *XRCC1* polymorphisms are listed in the Ensembl database, of which the most extensively studied are the genetic changes in exon 6 (Arg194Trp) and in exon 10 (Arg399Gln). The frequency of variant 194Trp is very low in Caucasians and African Americans (5%-11%), while the frequency of variant 399Gln ranges from 32 to 48% in Caucasians (Hu *et al.*, 2005). These changes in conserved protein sites may alter the base excision repair capacity, increasing the susceptibility to adverse health conditions, including cancer (Hung *et al.*, 2005).

In the present study, we estimated the frequencies of Arg194Trp and Arg399Gln in the *XRCC1* gene in healthy individuals and also in women at high risk of breast cancer due to family history (FH), from Rio de Janeiro, in order to analyze possible differences between these groups regarding the distribution of *XRCC1* variants.

Peripheral blood samples were collected from 418 healthy individuals from the general population (population sample) undergoing routine medical tests at the Pedro Ernesto University Hospital, in the city of Rio de Janeiro, and from 104 women participating in the “Breast Cancer and Genetics Project – National DNA Bank” (BNDNA) of the Fernandes Figueira Institute-FIOCRUZ, located in the same city. This DNA bank contains genomic DNA from no consanguineous women who reported breast cancer in first- and/or second-degree relatives (women with a family history of breast cancer). The control group consisted of 240 individuals (72 men and 168 women) taken from the population sample who had no reported case of breast or ovary cancer in the family and were not affected by breast or any other type of cancer. All the study subjects were recruited between 1996 and 2001, and epidemiological data were obtained by standardized questionnaires. Based on phenotype characteristics, the individuals were classified into whites and non-whites (mulattoes and blacks). Written informed consent was obtained from each subject. This study was approved by the Ethics Committees of the Pedro Ernesto University Hospital and the Fernandes Figueira Institute. Genomic DNA was prepared from the blood samples by proteinase K digestion, according to standard methods (Sambrook *et al.*, 1989). Genotyping of the *XRCC1* polymorphisms was performed as described by Deligezer and Dalay (2004). Some randomly selected samples were genotyped by direct sequencing (ABI Prism Sequence Detection System – Applied Biosystems) to confirm the results. Chi-square or Fisher's exact test were applied to compare the different groups with regard to genotype frequencies. Statistical analysis was performed using the GraphPad Instat 3 software (GraphPad Software, San Diego, CA); the probability level (p) of less than 0.05 was used as significance criterion.

The characteristics of the study groups “Population Sample” (n = 418), “Women with family history (FH) of breast cancer” (n = 104), and “Controls” (n = 240) are presented in Table 1. The mean age of the Population Sample was 49.7 ± 16.4 years, and its ethnic composition was 51% whites and 46% non-whites. All of them were inhabitants of the metropolitan area of Rio de Janeiro for at least 6 months. The group of FH women was slightly younger than the controls (mean age: 44.4 ± 13.1 and 49.3 ± 16.4 years, respectively). A significant difference (p < 0.05) with respect to ethnicity was observed: the number of white individuals was higher in the FH women compared to controls. It is important to note that 57% of the women of the FH group reported at least one first-degree relative affected by breast cancer, about 60% of which were diagnosed before the age of 50**.** Five women with a family history of breast cancer had breast cancer themselves at the time of blood collection. The frequency of the *XRCC1* polymorphism in the Population Sample is presented in Table 2, showing that the Arg/Arg genotypes in both positions (194 and 399) were the most frequent. The homozygous 194Trp genotype is extremely rare and was detected in only two individuals. When this group was stratified by ethnicity (whites and non-whites), we observed that the heterozygous genotype Arg/Trp for codon 194 and the homozygous genotype Gln/Gln for codon 399 were more prevalent in whites compared to non-whites. However, these differences were not statistically significant. Analysis of a possible intragenic association between codon 194 and codon 399 alleles showed that the wild-type (Arg194/Arg399) and the combination of Arg194 and 399Gln alleles were equally distributed in the Population Sample (43.5% and 44.0%, respectively). In this analysis, we did not observe any statistically significant difference between whites and non-whites (Table 3). In order to evaluate the possible contribution of the *XRCC1* polymorphisms to breast cancer susceptibility, we analyzed the genotype distribution in a group of women with an FH of breast cancer. Thus, the frequencies were determined in this particular group and compared with the genotype distribution observed in the controls. Since the frequency of *XRCC1* polymorphisms does not vary according to gender (male x female: p = 0.80 for codon 194; p = 0.15 for codon 399), the control group was composed of both men and women from the population sample with no previous cases of breast or ovary cancer in the family. The distribution of *XRCC1* polymorphisms was similar in both groups, although women with an FH of breast cancer showed a higher percentage of genotypes presenting the Trip allele (Arg/Trp + Trp/Trp) than the controls (19.3% and 13.3%, respectively). This difference was more noticeable when we considered only women reporting at least one affected first-degree relative: 22% x 13.3% in controls. On the other hand, the Gln allele for codon 399 was more frequent in the control group. However, these differences were not statistically significant (Table 4). Women reporting only second-degree relatives affected by breast cancer showed a similar genotype distribution and allele frequencies for both *XRCC1* variants compared to the control group. In the ethnicity analysis, the distribution of polymorphisms Arg194Trp and Arg399Gln in women with FH and in controls was similar in whites and non-whites (whites: p = 0.08 for codon 194; p = 0.44 for codon 399; non-whites: p = 1.00 for codon 194; p = 0.34 for codon 399**).** The intragenic association study did not show any statistically significant difference concerning the frequency of a particular genotype combination for both polymorphic variants when women with an affected first-degree relative and controls were compared (194Trp/Arg399: p = 0.49; Arg194/399Gln: p = 0.33; 194Trp/399Gln: p = 0.26. Reference: Arg194/Arg399). All genotype distributions of the *XRCC1* gene were in Hardy-Weinberg equilibrium.

The XRCC1 protein is considered an essential part of both the single-strand break repair and the base excision repair systems (Cappelli *et al.*, 1997). Here, we studied two *XRCC1* polymorphisms: Arg194Trp, located in exon 6, and Arg399Gln, located in exon 10. Both substitutions produced structural changes in the protein molecule, probably altering its biological activity and consequently affecting the DNA repair efficiency. These observations led us to realize the importance of determining the distribution of these genetic variants in our population. Brazil has a large territory and the differences in colonization among different geographical regions resulted in different levels of miscegenation (Alves-Silva *et al.*, 2000). So far, four papers have described the *XRCC1* genotype distribution in Brazilian populations, all of them from the State of São Paulo (Rossit *et al.*, 2002; Duarte *et al.*, 2005; Dufloth *et al.*, 2005; Canalle *et al.*, 2006). The distribution of both polymorphisms found by us in a population sample from Rio de Janeiro was similar to that found in São Paulo, the wild-type genotypes being the most frequent. Concerning ethnicity, the allele frequency for the 194Trp variant in Euro-descendents (whites) from Rio de Janeiro was 0.08 and in Afro-descendents (non-whites) 0.06 (Table 2). These results are in agreement with those observed in American and European Caucasians (0.05-0.09) and in African Americans (0.05-0.11) (Lunn *et al.*, 1999; David-Beabes and London, 2001; Smith *et a*l., 2003; Hu *et al.*, 2005; Pachkowski *et al.*, 2006). The frequency of the 399Gln variant allele was of 0.30 in Brazilians of European descent, quite similar to the frequencies reported for American Caucasians (0.32-0.37) and European Caucasians (0.32-0.48) (Lunn *et al.*, 1999; David-Beabes and London, 2001; Matullo *et al.*, 2001; Duell *et al.*, 2002; Smith *et a*l., 2003; Hu *et al.*, 2005; Pachkowski *et al.*, 2006). In the Brazilians of African descent, the frequency of the 399Gln allele was 0.25, about the same (0.27) as observed by Canalle *et al.* (2006) for Afro-descendents from São Paulo (Brazil), but higher than that observed in African Americans (0.14-0.18) (Lunn *et al.*, 1999; David-Beabes and London, 2001; Duell *et al.*, 2002; Hu *et al.*, 2005; Pachkowski *et al.*, 2006). The intragenic association analysis showed that the most frequent combinations were the wild-type Arg194/Arg399 and the genotype with one variant Arg194/399Gln (43.5% and 44.0%, respectively), revealing the high frequency of the Gln allele in the Brazilian population. It remains, however, to be clarified which are the reasons why the State of Rio de Janeiro is the one with the highest number of breast cancer cases in Brazil. Family aggregation of this disease is also largely observed, which led us to search for clues about genetic susceptibility. Although hereditary breast cancer is commonly associated with high-penetrance genes such as *BRCA1*/2 and *TP53* (Walsh *et al.*, 2006), a clear risk genotype for the most part of the breast cancer families has not yet been described (Antoniou and Easton, 2006). In an approach distinct from the classical case-control association studies, we compared the distribution of the most studied variants of the *XRCC1* gene in women reporting FH of breast cancer with the distribution in individuals without reported breast cancer cases in the family (controls). No statistical differences were observed in the genotype distributions or in the intragenic interactions of polymorphisms Arg194Trp and Arg399Gln. So, according to our data, these *XRCC1* gene variants do not appear to have any impact on breast cancer susceptibility in the analyzed population. To elucidate the role of *XRCC1* variants and cancer risk, several case-control studies have been conducted, but the results are inconsistent. Two meta-analysis studies described the 194Trp variant as being related to a decrease in cancer risk (Goode *et al.*, 2002; Hu *et al.*, 2005), but Chacko *et al.* (2005) observed an association between allele 194Trp and the risk of breast cancer in women from India. However, a recent meta-analysis performed with a large number of cases from the United States (Zhang *et al.*, 2006) did not find any relation between the presence of either the *XRCC1* Arg194Trp or the Arg399Gln polymorphisms, among other DNA repair genes, and risk of breast cancer. Repair genes are unequivocally important in the process of breast cancer susceptibility but, as several repair systems may superpose and act together, a clear picture in case-control studies is very difficult to achieve. In spite of our limited sample size, we found the same trend described in previous studies on breast cancer patients with an FH (Smith *et al.*, 2003; Dufloth *et al.*, 2005; Costa *et al.*, 2007). Further studies with larger samples are needed to elucidate the role of these *XRCC1* gene variants in the genetic susceptibility to breast cancer.

## Figures and Tables

**Table 1:** Descriptive characteristics of the population sample, women with family history (FH) of breast cancer and controls.

Characteristics	Population sample (n = 418)	Women with FH (n = 104)	Control (n = 240)
Age (years)	49.7 ± 16.4	44.4 ± 13.1	49.3 ± 16.2

Ethnicity, n (%)			

Whites	214 (51.0)	65 (62.0)	122 (51.0)
Non-whites	190 (46.0)	35 (34.0)	118 (49.0)
Missing data	14 (3.0)	4 (4.0)	0

Classification by degree of affected relatives^1^, n (%)			

First		59 (57.0)	
Second		45 (43.0)	

^1^First-degree relative: at least one first-degree relative affected by breast cancer; second- degree: at least one second-degree relative affected by breast cancer and no affected first-degree relative.

**Table 2:** Frequency of polymorphisms *XRCC1*-Arg194Trp and *XRCC1*-Arg399Gln in the population sample and distribution according to ethnicity.

Genotype *XRCC1*^1^	Population sample (n = 418) n (%)	White (n = 214) n (%)	Non-White (n = 190) n (%)	p-value^2^
Arg194Trp				
Arg/Arg	366 (87.6)	182 (85.0)	170 (89.5)	
Arg/Trp	50 (12.0)	31 (14.5)	19 (10.0)	
Trp/Trp	2 (0.5)	1 (0.5)	1 (0.5)	
Arg/Trp + Trp/Trp	52 (12.5)	32 (15.0)	20 (10.5)	0.23
Trp-allele frequency	0.07	0.08	0.06	

Arg399Gln				
Arg/Arg	223 (53.4)	109 (50.9)	106 (55.8)	
Arg/Gln	159 (38.0)	82 (38.3)	73 (38.4)	
Gln/Gln	36 (8.6)	23 (10.8)	11 (5.8)	0.18
Arg/Gln + Gln/Gln	195 (46.6)	105 (49.1)	84 (44.2)	
Gln-allele frequency	0.28	0.30	0.25	

^1^Missing data - 14 (3.3%) individuals of the population sample could not be classified according to ethnic group. ^2^Chi-square or Fisher's exact test.

**Table 3:** Intragenic association of the *XRCC1* polymorphisms (Population sample).

Exon 6 Codon 194	Exon 10 Codon 399	Population sample n (%)	White n (%)	Non-White n (%)	p-Value^1^
All wild-type genotypes					
Arg	Arg	182 (43.5)	84 (39.3)	90 (47.4)	

One variant polymorphism					
Trp	Arg	41 (9.8)	25 (11.7)	16 (8.4)	0.17
Arg	Gln	184 (44.0)	98 (45.8)	80 (42.1)	0.24

Two variant polymorphisms					
Trp	Gln	11 (2.7)	7 (3.2)	4 (2.1)	0.36

^1^Fisher's exact test (the wild-type genotypes were used as reference).

**Table 4:** Allele and genotype frequencies of polymorphisms *XRCC1*-Arg194Trp and *XRCC1*-Arg399Gln in women reporting a family history (FH) of breast cancer in first-degree relatives and controls.

Genotype *XRCC1*	Women with FH in first-degree relatives n (%)	Controls n (%)	OR (95%CI)^1^
Arg194Trp			
Arg/Arg	46 (78.0)	208 (86.6)	1.0 (reference)
Arg/Trp	12 (20.3)	32 (13.4)	1.70 (0.81-3.54)
Trp/Trp	1 (1.7)	0	ND^2^
Arg/Trp + Trp/Trp	13 (22.0)	32 (13.3)	1.84 (0.89-3.77)
Trp allele frequency	0.12	0.07	

Arg399Gln			
Arg/Arg	33 (55.9)	120 (48.8)	1.0 (reference)
Arg/Gln	25 (42.4)	103 (41.9)	0.88 (0.49-1.58)
Gln/Gln	1 (1.7)	23 (9.3)	0.16 (0.02-1.22)
Arg/Gln + Gln/Gln	26 (44.1)	126 (51.2)	0.75 (0.42-1.33)
Gln allele frequency	0.23	0.30	

^1^Fisher's exact test. ^2^ND not determined due to small sample size in the category.
